# Loss of *Glis3* causes dysregulation of retrotransposon silencing and germ cell demise in fetal mouse testis

**DOI:** 10.1038/s41598-018-27843-x

**Published:** 2018-06-25

**Authors:** Erica K. Ungewitter, Emmi Rotgers, Hong Soon Kang, Kristin Lichti-Kaiser, Leping Li, Sara A. Grimm, Anton M. Jetten, Humphrey H.-C. Yao

**Affiliations:** 1 Reproductive Developmental Biology Laboratory, National Institute of Environmental Health Sciences, Durham, NC USA; 2 Immunity, Inflammation & Disease Laboratory, National Institute of Environmental Health Sciences, Durham, NC USA; 3 Biostatistics & Computational Biology Branch, National Institute of Environmental Health Sciences, Durham, NC USA; 4 Integrative Bioinformatics Support Group, National Institute of Environmental Health Sciences, Durham, NC USA

## Abstract

Fetal germ cell development is regulated by an elaborate combination of cell-extrinsic and cell-intrinsic signals. Here we identify a novel role for the Krüppel-like transcription factor Gli-Similar 3 (*Glis3*) in male germ cell development in the mouse embryos. *Glis3* is expressed in male germ cells during the brief window of time prior to initiation of piRNA-dependent retrotransposon surveillance. Disruption of *Glis3* function led to a widespread reduction in retrotransposon silencing factors, aberrant retrotransposon expression and pronounced germ cell loss. Experimental induction of precocious *Glis3* expression *in vivo* before its normal expression resulted in premature expression of several piRNA pathway members, suggesting that GLIS3 is necessary for the activation of the retrotransposon silencing programs. Our findings reveal an unexpected role for GLIS3 in the development of male germ cells and point to a central role for GLIS3 in the control of retrotransposon silencing in the fetal germline.

## Introduction

Germ cells are a specialized population of cells that gives rise to gametes and, upon conception, form a continuous chain of genetic information between generations. In mice, primordial germ cells (PGCs) are specified at early gastrulation, around embryonic day (E) 6.25, then undergo a rapid burst of proliferation and migration to reach the developing gonads around E10.5^1^. PGCs also undergo extensive epigenetic remodeling – including global DNA demethylation, chromatin reorganization, and imprint erasure - during this phase of development^2^. These epigenetic changes are vital to reset methylation marks as the germline enters a new generation but leave PGCs vulnerable to genetic damage from transposable DNA elements.

It is imperative for germ cells to protect the integrity of their ‘immortal’ genomes and they have developed several unique mechanisms to do so, which include global transcriptional repression, chromatin state alteration and expression of germline specific PIWI/piRNA factors^3^. Mis-regulation and defects in these mechanisms such as in mice lacking components of the PIWI/piRNA pathway, including Piwi-like 1(*Piwil1*), *Piwil2*, *Piwil4*, *Vasa*, *Dnmt3l*, *Hormad1*, *Prmt5* and others^4–10^ led to germ cell loss and infertility. In all of these examples, germ cells are lost in postnatal life, concordant with the transition from germ cell (gonocyte) to spermatogonial stem cell. Fetal loss of male germ cells, by contrast, is not common and the mechanism(s) underlying this process are not well understood.

In this study, we set out to characterize the role of a testis-enriched transcription factor, GLIS3^11^, in male germ cell development in mouse embryos. GLIS3 is a member of the GLI-Similar (GLIS) family of Krüppel-like transcription factors, named for their high degree of sequence homology to the zinc finger domains of the Gli/Zic proteins^12^. *Glis3* is highly expressed in mid-gestation kidney and pancreas and has been shown to be essential for the development of these organs^13^. Homozygotes of a weaker *Glis3* mutant allele (*Glis3*^*KO2*^) have defects in spermatogonia differentiation and proliferation during early postnatal testis development in the mouse^14^. Testes from this hypomorphic *Glis3* mutant strain appeared normal at birth but contain little to no germ cells by 8 weeks of age. Genes associated with undifferentiated spermatogonia, including *Ret*, *Lhx1* and *Pouf51*, were suppressed in the neonatal testis of this *Glis3* mutant line, suggesting that normal GLIS3 function is important for transition from germ cell to spermatogonial stem cell during early spermatogenesis^14^.

Given the unique pattern of *Glis3* expression in embryonic testis, we hypothesized that it also plays an important role during fetal testis development. In this study, we analyzed the testis phenotypes in a nonfunctional *Glis3* knockout mouse and examined its role in male fetal germ cell survival and its potential involvement in retrotransposon silencing programs.

## Results

### *Glis3* is expressed predominantly in germ cells

**Figure 1:**
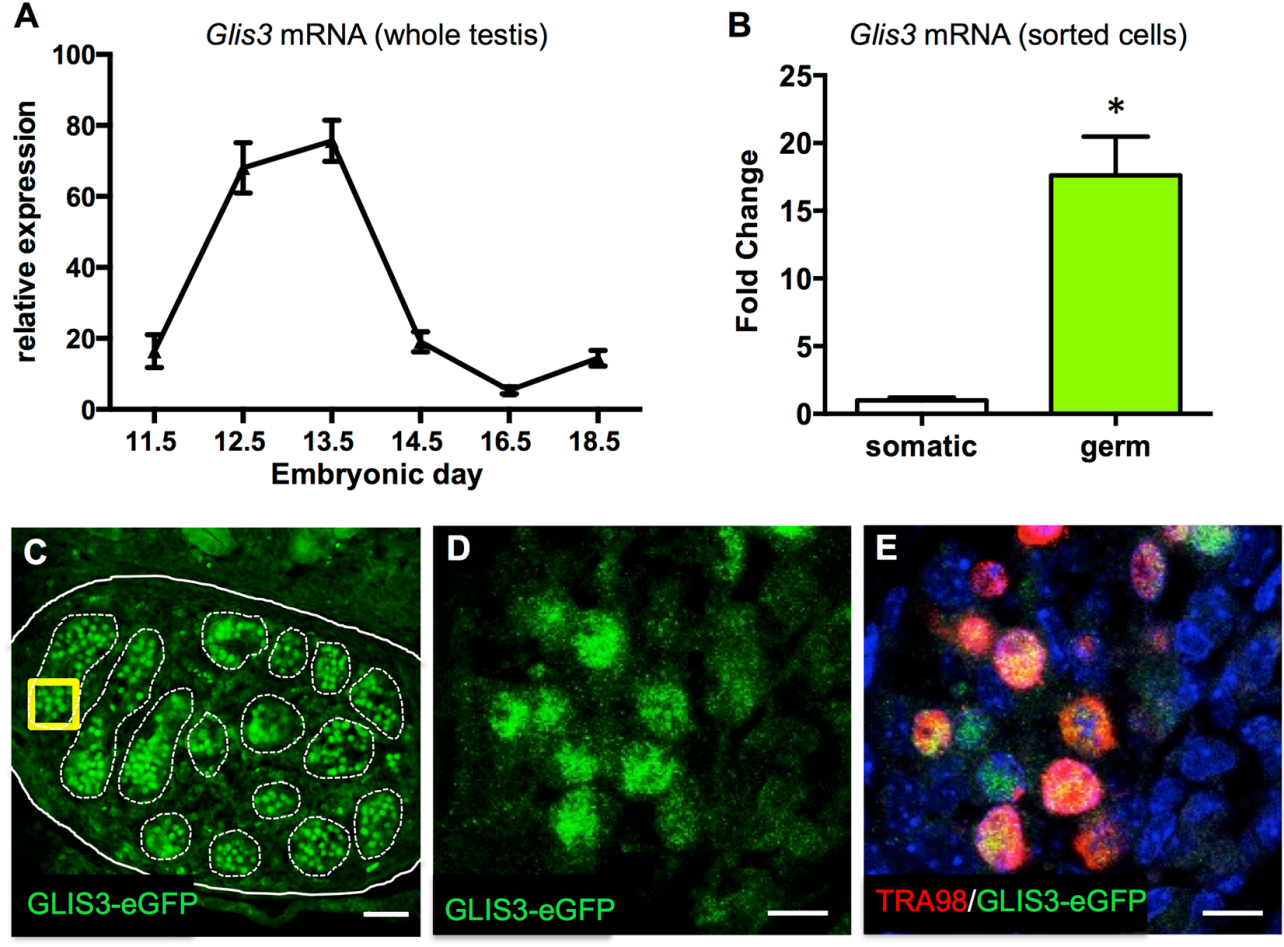
*Glis3* is expressed in fetal germ cells in the testis. (**A**) qPCR analysis of *Glis3* mRNA levels in wild-type CD-1 mouse fetal testes from E11.5 to E18.5. (**B**) qPCR analysis of *Glis3* mRNA in FACS-isolated somatic or germ cell populations from E13.5 Oct4-eGFP testes. Expression levels of Oct4-eGFP negative cells are set as 1. *p < 0.005 by Student’s T test. Values in both graphs are presented as mean ± s.e.m.; N = 3–4 biological replicates. (**C–E**) Immunofluorescence for Glis3-eGFP (green) in E13.5 testis from the *Glis3-eGFP* reporter mouse line. White dotted and solid lines outline the testis cords and fetal testis, respectively. The yellow inset from panel C is shown at greater magnification in panels D and E. (**E**) Immunohistochemistry for *Glis3-eGFP* (green), germ cells (TRA98; red), and DNA (DAPI; blue). Scale bars: (**C**) 50 μm, (**D,E**) 10 μm.

The first goal of our study was to precisely characterize the expression pattern of *Glis3* during fetal testis development. Attempts to generate specific antibodies against mouse GLIS3 have thus far been unsuccessful. We therefore turned our focus to mRNA levels using quantitative real-time PCR (qPCR). In wild-type fetal testes, *Glis3* mRNA levels rose sharply around E12.5 and returned to baseline by E14.5 (Fig. 1A). To determine the cellular source of *Glis3* in fetal testes, we separated somatic and germ cell fractions from E13.5 *Oct4-eGFP* testis, in which germ cells are marked by enhanced GFP fluorescence, by FACS (Supplemental Fig. 1). *Glis3* mRNA was predominantly detected in male germ cells with low expression in the somatic cells, which is consistent with *Glis3* expression pattern described in isolated fetal germ cells at E11.5 to E13.5 (Fig. 1B)^15^. The germ cell-specific expression of *Glis3* was further confirmed with a knock-in mouse line, in which the endogenous GLIS3 protein is fused to an enhanced GFP protein (*Glis3-eGFP*). In E13.5 fetal testes, *Glis3-eGFP* was found predominantly in the germ cells located inside testis cords, overlapping with the germ cell marker TRA98 (Fig. 1C–E).

### *Glis3* is essential for the development and survival of male fetal germ cells

**Figure 2:**
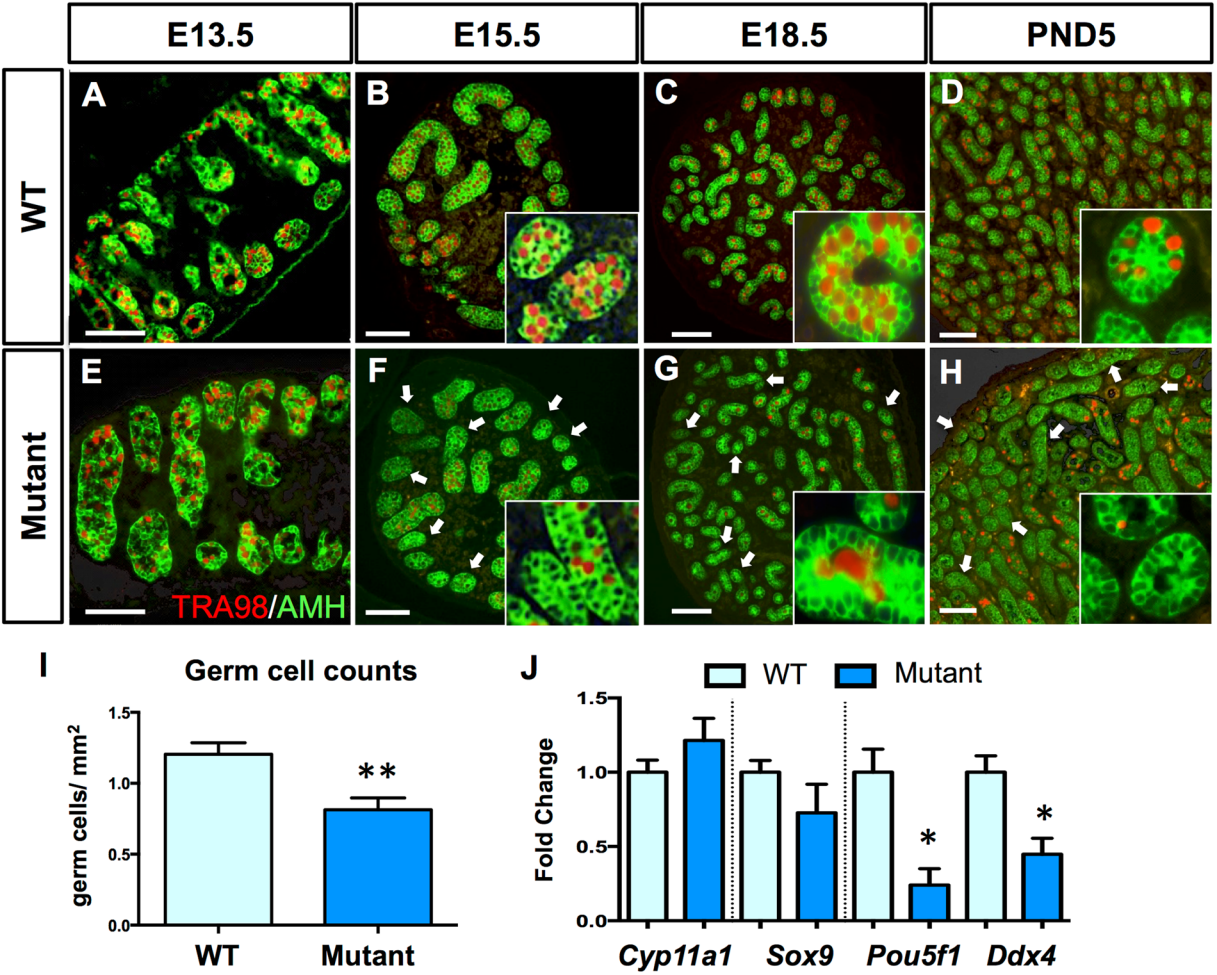
Fetal male germ cells are progressively lost in *Glis3* mutant testes. (**A–H**) Immunohistochemistry for the Sertoli cell marker AMH (green) and germ cell marker TRA98 (red), in wild type (WT) and *Glis3*^*zf5/zf5*^ mutant testes at E13.5, E15.5, E18.5, and PND5. Arrows indicate testis cords devoid of TRA98-positive germ cells. Insets are higher magnification regions of (**B-D,F-H**). Scale bar = 50 μm. (**I**) Quantification of germ cell number per square mm in WT and mutant testes at E15.5. *p < 0.05, **p < 0.01 by Student’s T test. (**J**) qPCR analysis of mRNA levels of somatic cell markers (*Sox9* for Sertoli cells and *Cyp11a* for Leydig cells) and germ cell markers (*Ddx4* and *Oct4*) in WT and mutant testes at E15.5. Expression levels of WT samples are set as 1. Values in all graphs are presented as mean ± s.e.m.; N = 5–6 biological replicates per genotype (**I**) or 3–4 replicates (**J**).

The window of peak *Glis3* expression (E12.5–14.5) coincides with two critical events in early germ cell development: the choice between meiotic entry versus mitotic arrest and genome-wide demethylation. To determine the functional significance of *Glis3*, we examined testis development from the global *Glis3* null mutation strain (*Glis3*^*zf5*^, hereafter referred to as *Glis3* mutant) from E13.5 to postnatal day (PND) 5, the latest that mutant animals can survive. *Glis3*^*zf5/zf5*^ homozygotes develop severe pancreatic and thyroid phenotypes that cause death at or before PND7^13^, in contrast to the weaker *Glis3*^*KO2*^ mutant strain, which survives until approximately 2 months of age^14^. Gross morphological analysis revealed no overt changes in *Glis3*^*zf5/zf5*^ mutant testes at these stages of development. Morphogenesis of the testis structures and somatic cell differentiation were not affected based on the immunostaining of AMH, a marker of Sertoli cell differentiation (Fig. 2A–H) and expression of *Cyp11a1*, a marker for Leydig cell differentiation (Fig. 2J). At E13.5, numbers of germ cells marked with TRA98 were similar between the control and *Glis3*^*zf5/zf5*^ mutant testes, indicating that germ cell specification and migration occurred normally (Fig. 2A,E). However, starting at E15.5, a significant reduction in TRA98- positive germ cells became apparent in mutant testes (arrows in Fig. 2A) and by PND5, many mutant testis cords were devoid of any germ cells (Fig. 2H). At E15.5, we observed a 40% reduction in germ cell numbers in mutant testes (Fig. 2I). The decrease in germ cell numbers in mutant testes at E15.5 was in parallel with a significant reduction in expression of the germ cell-specific genes *Oct4* and *Vasa* (Fig. 2J). A similar phenotype was observed at PND5. We were not able to examine the germ cell phenotype beyond PND5 because the *Glis3* mutant animals did not survive past PND5. These results indicate that *Glis3* is highly enriched in the germ cells, and is required for germline survival in male fetal testes. Somatic cell populations were not altered in mutant testes, suggesting that germ cell loss is not a consequence of deficiencies in somatic cell differentiation.

### Identifying the mechanism(s) of GLIS3 action in the male germline

**Figure 3:**
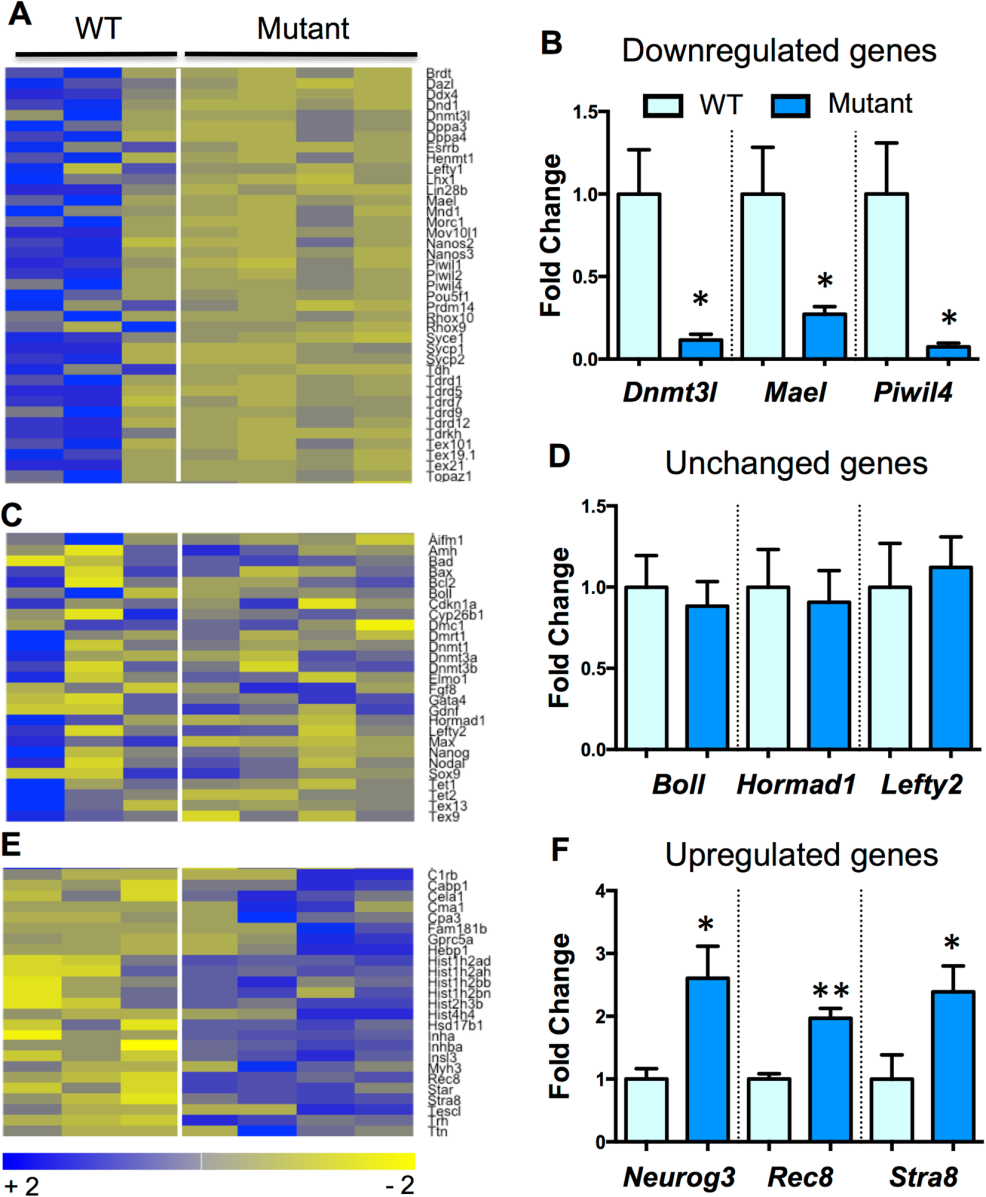
Germ cell development pathways are selectively dysregulated in the absence of functional *Glis3*. (**A,C,E**) Heatmaps, generated from RNA-Seq analyses, illustrate changes in several germ cell-related genes in wild-type (WT) vs. *Glis3*^*zf5/zf5*^ mutant testes at E14.5. (**A**) downregulated genes, with reduced expression in mutant versus wild-type samples; (**C**) genes unchanged between groups; and (**E**) upregulated genes, with increased expression in mutant testes. Each sample column represents a pair of testes from a single embryo. Three to four biological replicates from each genotype were analyzed using Cuffdiff, resulting in differential expression of 673 unique genes (FDR adjusted p < 0.05). (**B,D,F**) qPCR confirmation of genes identified by RNA-Seq. *p < 0.05; **p < 0.005 by Student’s T test. Values in all graphs are presented as mean ± s.e.m; expression levels of WT samples are set as 1. N = 5–8 biological replicates per genotype.

To identify the mechanisms by which GLIS3 controls male germ cell survival, we performed mRNA sequencing (RNA-Seq) on whole testes of wild type and *Glis3* mutant embryos at E14.5, before the onset of germ cell loss in mutant testes. At E14.5, germ cell numbers in the *Glis3* mutant testes (100 ± 40 germ cells/mm^2^) were not different from those in the wild-type testes (160 ± 50 vs. germ cells/mm^2^). GO biological process analysis of these data (Supplemental Fig. 2) revealed that piRNA biosynthesis, catabolism and metabolism were the most affected processes in mutant testes. Multiple piRNA pathway components, including *Ddx4*, all three of the mammalian PIWI-like genes (*Piwil1*, *Piwil2*, and *Piwil4*), Maelstrom *(Mael)*, and several Tudor-domain containing genes (*Tdrd1*, *Tdrd5*, and *Tdrd9*) were profoundly reduced in *Glis3* mutant testes (Fig. 3A; qPCR validation of select genes shown in 3B). The germ cell specific DNA methyltransferase co-factor *Dnmt3l* was also significantly reduced in mutant testes, as was MORC family CW-type zinc finger 1 *(Morc1)*, a repressor of retrotransposon activity^16^. Individual loss of each of these genes is known to cause ectopic retrotransposon activation, meiosis prophase I germ cell arrest, and germ cell loss that appears postnatally^4–6,16–21^.

Several genes implicated in spermatogenesis, including the zinc-finger motif-containing RNA-binding proteins *Nanos2* and *Nanos3* were also significantly decreased in *Glis3* mutant testes. XY germ cells from mice deficient in Nanos2 upregulate *Stimulated by retinoic acid 8* (*Stra8*) expression and enter meiosis precociously (Tsuda *et al*. 2003, Suzuki *et al*. 2008a). Although *Stra8* mRNA was elevated in *Glis3* mutant testes (see below), STRA8 protein was not expressed at E15.5 (Supplemental Fig. 3), indicating that germ cell loss in *Glis3* mutants is not due to NANOS2-mediated meiotic initiation. XY germ cells from mice deficient in N*anos3* are lost via Bax-dependent apoptosis prior to colonization of the gonads (beginning E9.0) (Suzuki *et al*. 2008b). *Glis3* mutant testes did not contain any TUNEL positive cells at E15.5 (data not shown) and contained normal cell numbers at E13.5 (Fig. 2), indicating that NANOS3-mediated apoptosis is not a major contributor to the germ cell loss phenotype of *Glis3* mutant XY germ cells. Finally, several germ cell-specific genes including Boule-like RNA-binding protein (*Boll*), HORMA domain containing 1 (*Hormad1*), and Left-right determination factor 2 (*Lefty2*) were unchanged in mutant testes (Fig. 3C,D), indicating that changes in germ cell related genes (piRNA pathway components or *Nanos* genes) are not the result of general germ cell deficiency.

A subset of germ cell development genes, including the meiosis related genes *Stimulated by retinoic acid 8* (*Stra8*) and *REC8 meiotic recombination protein* (*Rec8*) were significantly upregulated (Fig. 3E,F) in *Glis3* mutant testes. High levels of *Stra8* are typically found in germ cells that have initiated meiosis^22^. To determine if *Glis3* mutant germ cells had entered meiosis precociously, we performed immunostaining for STRA8 and the meiotic synaptonemal protein complex 3 (SYCP3). Comparable to wild type testes, *Glis3* mutant testes did not contain any STRA8 or SCP3 positive cells (Supplemental Fig. 3), indicating that, despite ectopic expression of some meiotic genes in the absence of functional GLIS3, male germ cells do not enter meiosis. Changes in germ cell-specific gene expression were not secondary to defects in somatic cell differentiation, based on the fact that genes known to control somatic cell differentiation in the testis (for example *Amh*, *Gata4*, and *Sox9*) and germ cell meiosis (*Cyp26b1)* were not altered (Fig. 3C), and somatic cell morphology was normal in *Glis3* mutant testes (Fig. 2). These results reveal an unexpected reduction in the expression of retrotransposon-silencing PIWI/piRNA pathway members and *Nanos* RNA binding factors in the absence of functional GLIS3.

### Transposable element activation in testes lacking functional Glis3

**Figure 4:**
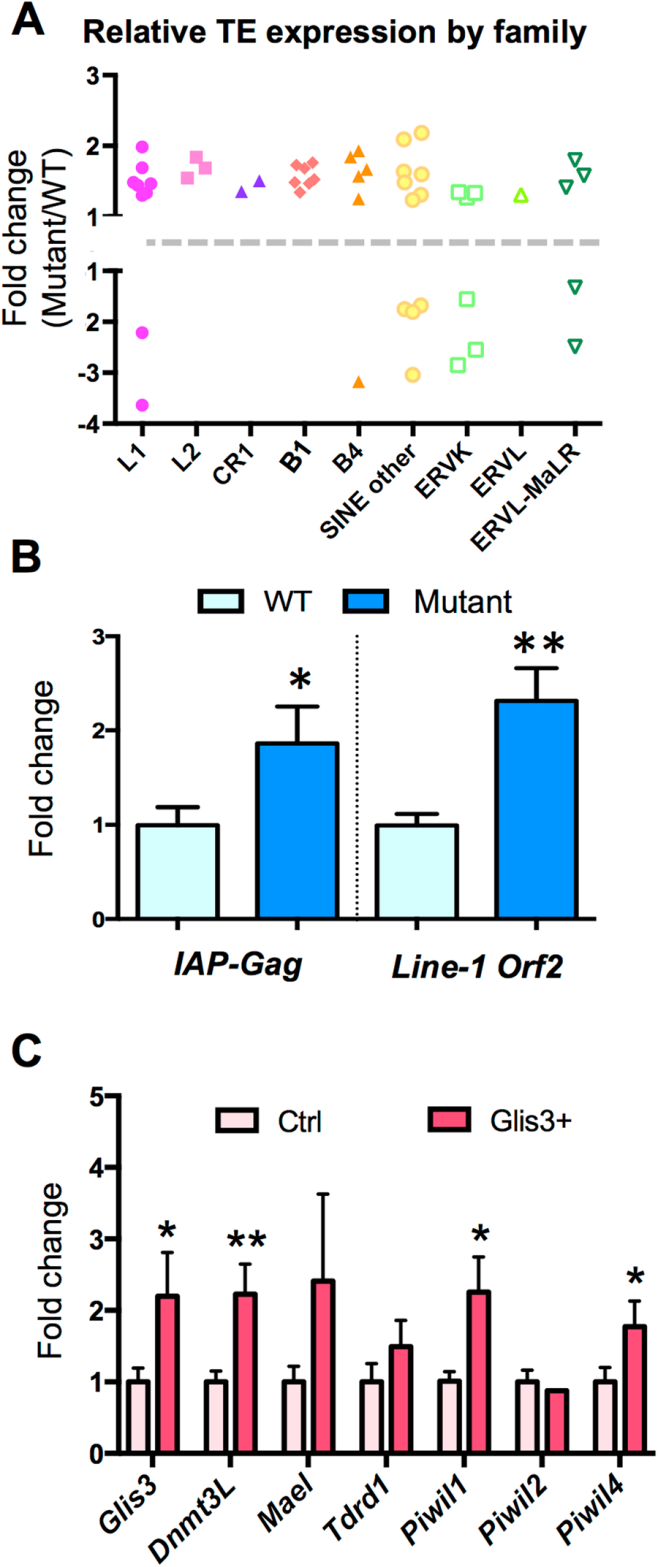
(**A**) Relative expression of unique retrotransposon loci in *Glis3*^*zf5/zf5*^ mutant relative to WT testes at E14.5. Columns separate retrotransposons by subfamily, with each symbol representing the fold change of an individual retrotransposon. Pink/purple symbols were assigned to loci from the LINE subfamily, orange/yellow symbols to SINE elements, and green symbols to LTR elements. (**B**) qPCR analysis of the Gag polyprotein of *IAP* and open reading frame (ORF) 2 of *Line-1* in wild-type (WT, pale blue) and mutant testes (blue) at E15.5. *p < 0.05; **p < 0.001 by Student’s T test. Values are presented as mean ± s.e.m; expression levels of WT samples are set as 1. N = 6–7 biological replicates per genotype. (**C**) The expression of *Glis3* was ectopically and precociously induced in male germ cells of *Dppa3-creER; R26-Glis3* mice by tamoxifen administration on E10.5 and E11.5. The expression of *Glis3* mRNA and a set of retrotransposon silencing genes (*Dnmt3l*, *Mael*, *Tdrd1*, *Piwil1*, *Piwil2*, and *Piwil4*) was analyzed in control (pale pink) and R26-Glis3 + (pink) E12.5 testes using qPCR and normalized to *Gapdh*. Unpaired t-test with Welch’s correction was used as a statistical test. *p < 0.05, **p < 0.01. n = 11–14 per group.

The widespread reduction in expression of retrotransposon-silencing pathway components in *Glis3* mutant testes suggested a compromised function of this system, which would consequently result in increased retrotransposon mRNA expression. To determine if retrotransposon expression was affected in Glis3 deficient testes, we assessed transcriptome-wide changes in retrotransposon expression in our RNA-Seq dataset using RepeatMasker annotations. We discovered significant changes in a subset of elements from all three major retrotransposon classes: long interspersed nucleotide element (LINE), short interspersed nucleotide element (SINE), and long terminal repeats (LTR or ERV) classes (Fig. 4A). While most affected retrotransposons exhibited increased expression in mutant relative to wild-type testes (Fig. 4A, ratio >1), reduced expression of several elements was also observed (Fig. 4A, ratio <1). We confirmed the ectopic activation of retrotransposons known to be targets of the piRNA pathway, including the LTR intracisternal A particle (IAP) and LINE-1^4,6^ using qPCR. Both transcripts were significantly elevated in *Glis3* mutant testes at E15.5, the time when germ cell numbers begin to decrease (Fig. 4B). These data indicate that one of the normal functions of GLIS3 is to suppress expansion of select retrotransposons in the male germline immediately following the commitment of these cells to a male fate.

### Precocious induction of retrotransposon silencing factors following ectopic *Glis3* expression

If the normal role of GLIS3 is to promote the expression of factors necessary for retrotransposon silencing in male germ cells, then one would predict that ectopic upregulation of *Glis3* prior its normal induction should result in a premature upregulation of retrotransposon-silencing genes. In order to test this hypothesis, we generated a Rosa26 (R26)-driven Glis3 line, which allows us to induce ectopic *Glis3* expression with cell type-specific Cre recombinase (Supplemental Fig. 4A). By crossing the *R26-Glis3* to a tamoxifen-inducible germ cell-specific Cre recombinase line (*Dppa3-creER*)^23^, we produced a model that allows *Glis3* expression in germ cells after tamoxifen treatment. The increase of endogenous *Glis3* began at E12.5 (Fig. 1A). Therefore, to force a premature expression of *Glis3* in fetal germ cells, we injected tamoxifen to the dam carrying control embryos (*Dppa3-creER*-negative, *R26-Glis3*-positive) and *Glis3*+ mutant (*Dppa3-creER*-positive, *R26-Glis3*-positive) embryos on E10.5 and E11.5. qPCR analysis revealed that *Glis3* expression was significantly higher on E12.5 in the *Glis3*+ testes compared to control testes (Fig. 4C), indicating that a premature induction of *Glis3* expression was achieved. The retrotransposon-silencing genes *Dnmt3l*, *Piwil1*, *Piwil4* and *Mael* were also significantly increased in *Glis3*+ testes compared to controls, demonstrating that these genes are positively correlated with *Glis3* expression. The specific effect of *Glis3* on retrotransposon silencing genes is further supported by the fact that no significant changes in expression of the somatic cell gene *Wt1* or the generic germ cell gene *Oct4* were observed in *Glis3*+ testes (Supplemental Fig. 4B). Together these findings indicate that GLIS3 is sufficient for the induction of several retrotransposon silencing genes in male fetal germ cells.

## Discussion

In this study, we identified a novel and critical role for the transcription factor GLIS3 in the regulation of male fetal germ cell development and the induction of retrotransposon silencing pathways. *Glis3* mRNA in fetal mouse testes is expressed in the developing germ cells during a brief window of time from E12.5 to E13.5. The timing of *Glis3* expression in the male mouse germline coincides with several important developmental milestones: the commitment of these cells to a gender-specific developmental program, global demethylation of the germline and activation of the PIWI/piRNA retrotransposon silencing pathway. Global inactivation of *Glis3* did not significantly alter overall testis development, but induced a dramatic loss of male germ cells. Germ cell loss was first detected at E14.5 and was rapidly progressive; very few TRA98 positive germ cells remained at the end of our study, PND5, the latest that homozygous *Glis3* mutant animals can survive.

RNA-Seq analysis of mutant testes before the onset of germ cell loss (E14.5) identified dramatically reduced expression of PIWI/piRNA pathway factors and Nanos genes as well as increased expression of a subset of meiosis related genes. Although no signs of precocious meiotic entry were observed in *Glis3* mutant germs cells, we did find increased expression of multiple LINE and SINE transposon elements, confirming a general suppression of transposon silencing mechanisms in the absence of normal *Glis3*. We further characterized the role of *Glis3* in transposon silencing by forcing its expression in E10.5 germ cells *in vivo* before the normal rise of endogenous *Glis3* using a tamoxifen inducible R26-Glis3 model. We found that exogenous early onset of *Glis3* expression was sufficient to activate expression of critical PIWI/piRNA factors in the fetal male germ cells. The findings described herein identify a novel role for *Glis3* in the activation of transposon silencing systems in male fetal germ cells.

The progressive germ cell loss phenotype of *Glis3*^*zf5*^ mutant males is reminiscent of the germ cell defects described for retrotransposon silencing pathway members, including *Dnmt3l*, *Mael*, *Tdrd9*, *Tdrd1*, and all three of the mouse *Piwi-like* family members (*Miwi*, *Mili*, and *Miwi2*). Deletion of any single one these genes resulted in increased retrotransposon expression and meiotic arrest, which is coupled with germ cell loss in *Miwi2* and *Dnmt3l* mutants, mostly beginning around 14 days after birth^4–6,16–21,24^. The most significant difference between these mutant models and our *Glis3* mutant is that the germ cell loss phenotype in *Glis3* mutants occurs much earlier (postnatal vs. fetal). We hypothesize that the earlier onset of germ cell loss in *Glis3* mutants is the result of the global reduction of PIWI/piRNA pathway members, which was detected beginning ~E14.5. It is also possible that retrotransposon de-repression in *Glis3* mutant testes occurs via a (yet to be identified) piRNA-independent mechanism. DNA damage was not detected at the onset of the germ cell loss, but we cannot rule out its contribution to the phenotype.

The molecular mechanisms underlying gene transactivation by GLIS3 in male germ cells remain to be identified. The loss of male germ cells in the *Glis3* mutant is also accelerated compared to the germ cell phenotype of *Glis3KO2* mice, consistent with the hypomorphic description of the latter mutant allele^14,25^.

Previous studies in pancreatic and osteoblast progenitor cells have found three direct targets of GLIS3: *Fgf18*, *Ins2*, *and Ngn3*^25–30^. Via its regulation of these genes, GLIS3 has been implicated in differentiation of the osteoblast^31^ and pancreatic beta cell lineages^13^, respectively. Mice harboring *Glis3*^*zf5*^ null mutation (the same knockout model used in our study) exhibit polycystic kidney disease, hyperthyroidism, severe hyperglycemia and hypoinsulinemia, resulting in death within the first week of life^25^. Preliminary *in silico* analyses suggest that putative GLIS3 binding sites are found in the promoters of several genes misregulated in *Glis3* mutant testes (Supplemental Fig. 5 and Supplemental Table 1). These results point to a direct role for GLIS3 in the silencing of germ cell development genes, however *in vitro* promoter analyses are required to confirm these predictions.

In summary, we have discovered that *Glis3* is required for normal activation of retrotransposon silencing pathways in male fetal germ cells and is essential for their survival. This work advances our understanding of the factors guiding fetal germ cell development in mammals.

## Methods

### Animals

All animal studies were conducted in accordance with the NIH Guide for the Care and Use of Laboratory Animals and approved by the National Institute of Environmental Health Science (NIEHS) Animal Care and Use Committee. *Oct4-eGFP* (#008214) mice were purchased from the Jackson Laboratory and CD1 (#022) mice were purchased from Charles River laboratories. *Glis3*^*zf5*^ mutant mice were previously described;^13^ they harbor a 3.5 kb deletion in exon 4 and parts of introns 3 and 4, resulting in disruption of its DNA-binding capacity and impairment of GLIS3 transcriptional activation function. The *Glis3-eGFP* mouse strain is a knock-in model in which the full-length GLIS3 protein is fused to enhanced green fluorescent protein^32^. *R26-Glis3* (See Fig. S4A and description below) females were time-mated with *Dppa3-creER* (or *Dppa3-MERcreMER*)^23^ males. *Dppa3* is widely expressed in the pluripotent cells during early embryonic development, but later in development its expression is restricted to the primordial germ cells. Using the *Dppa3-creER* mouse line, Cre-recombinase can be activated with tamoxifen in the primordial germ cells from E9.5 onwards. For timed mating, female mice were housed with male mice overnight and checked for the presence of vaginal plug the next morning. Noon of the day when the vaginal plug was detected was considered embryonic day (E) 0.5. The day of birth was considered postnatal day 0 (PND0). All experiments were performed on at least three animals for each genotype. For Tamoxifen induction, 5 mg of Tamoxifen (cat# T5648, Sigma-Aldrich, St. Louis, MO, USA) diluted in corn oil was injected i.p. on E10.5 and E11.5. The embryos were collected on E12.5. For studying RNA expression, both of the testes were separated from the mesonephros and frozen in −80C. RNA was extracted using the Picopure kit according to manufacturer’s recommendations.

### Generation of *R26-Glis3* transgenic mouse line

In order to induce *Glis3* expression in a tissue specific manner, we generated a *Rosa-Glis3* mouse line (See Supplemental Figure S3). A *LoxP*-flank stop cassette is located downstream of the CAG promoter and *Glis3* coding sequence, preventing transcription from either the endogenous Rosa26 promoter or the CAG promoter. Following CRE-mediated excision of the stop cassette, a fusion transcript is made that contains the N-terminus HA-tagged, C-terminus FLAG-tagged, GLIS3 coding sequence (HA-GLIS3-FLAG) and dTomato coding sequence. Each transcript has its own ribosomal entry sequence and will produce two distinct proteins, not a fusion GLIS3-dTomato protein.

### Tissue Processing and Immunohistochemistry

For immunohistochemistry on frozen sections, testes were fixed in 4% paraformaldehyde in PBS at 4 ^o^C overnight, dehydrated through a sucrose gradient, embedded in OCT and cryosectioned at 10 µm increments. Testes collected after birth were embedded in paraffin wax using the following methods: samples were fixed in 4% paraformaldehyde in PBS at 4 ^o^C overnight, dehydrated through an ethanol gradient, and embedded in paraffin wax. 6-µm-thick sections were dewaxed and rehydrated in a series of alcohol just prior to immunostaining. All tissue sections (frozen or paraffin sectioned) were preincubated with 5% normal donkey serum in PBS for 1 hour, then incubated with either anti-AMH (1:500; sc-6886, Santa Cruz), anti-GFP (1:500; A1172, Life Technologies) or anti-TRA98 (1:1000, MBL international), in PBS-Triton X-100 solution with 5% normal donkey serum at 4 ^o^C overnight. The antibody-labeled tissue sections were then washed 3 times in PBS and incubated in the appropriate secondary antibody (1:500; Invitrogen) before a final 3 washes (with PBS) and mounting in Vector Mount with DAPI (Vector Labs). Slides were imaged under a Leica DMI4000 confocal microscope.

### Estimated germ cell counts

Whole E14.5 and E15.5 testes were prepared for frozen sections as described above, serial sectioned at 10 μm increments, immunostained for TRA98, and mounted with DAPI. Slides were scanned using the Aperio Scanscope FL Scanner (Aperio: Leica Biosystems Inc.). To produce representative germ cell counts, every sixth tissue section (60 μm increments) of individual testis samples was analyzed for both the number of TRA98 positive germ cells and total testis area (by DAPI) using Definiens Tissue Studio Software v3.0 (Definiens). Three to four biological replicates per genotype were analyzed with each containing 8–10 tissue sections. Germ cell number is calculated as the total number of TRA98 positive cells per square mm of testis tissue.

### Germ cell isolation

Germ cells were isolated from E13.5 *Oct4-eGFP* testes using a previously reported protocol for tissue dissociation and FACS^33^. Briefly, *Oct4-eGFP* testes from individual pups were dissected in PBS, and up to four pairs of testes were pooled for dissociation and sorting. Testes were enzymatically dissociated in PBS containing 1 mg/ml collagenase B (Roche), 1.2 U/ml Dispase II (Roche), and 5 U/ml DNase I (Sigma) at 37 °C for 20–30 minutes with shaking. Cells were further dissociated mechanically using a P200 pipette. After quenching of enzyme activities by addition of 2 mM EDTA, cells were pelleted for 5 min at 1000 g and resuspended in 2 mM EDTA in PBS. Cell-sorting was performed using a BD FACSAria II in the NIEHS Flow Cytometry Center.

### RNA extraction and gene expression analysis

Total RNA was isolated from frozen cells or tissues using the PicoPure RNA isolation kit (Arcturus) according to the manufacturer’s protocol. First-strand cDNA synthesis was performed using the Superscript II cDNA synthesis system using the manufacturer’s protocol (Invitrogen). The mRNA levels of genes were measured using either SYBR green (Applied Biosystems) or Taqman (Bio-rad) assays. Taqman probe and primer sequences used in qPCR are listed in Supplemental Material, Supplemental Table 2. Cycle threshold (Ct) values were obtained using the Bio-Rad CFX96TM Real-Time PCR Detection system. The relative fold change of each transcript was calculated using the mathematical model of Pfaffl^34^ and was normalized to *Mapk1* (Fig. 1A), or *Gapdh* (all other graphs) as an endogenous reference. All qPCR analyses were performed in duplicate, and the results were reported from at least three independent experiments. Results are presented as fold change calculated relative to WT controls. All values were expressed as mean ± SEM, and statistical analyses were performed using unpaired Student *t*-*test* with Welch’s correction and one-tailed p-value (GraphPad Prism). The sample sizes were 11 for the control testis and 14 for the *Glis3*+ testes.

### RNA-Sequencing library generation

RNA from a single pair of E14.5 testes (one biological replica) was extracted using the PicoPure extraction kit (Arcturus). Three or four biological replicas were used for each genotype. RNA-Seq libraries were constructed from ~0.3 μg total RNA after rRNA depletion using Ribo-Zero GOLD (Illumina). The Illumina TruSeq RNA Sample Prep V2 Kit was used according to manufacturer’s instructions except where noted. The poly-A selection step was omitted and random primers were used for first-strand synthesis. cDNAs were fragmented to ~275 bp using a Covaris E210. Amplification was performed using 16 cycles, which was optimized for the input amount and to minimize the chance of over-amplification. Unique barcode adapters were applied to each library. The pooled libraries were sequenced on multiple lanes of a HiSeq. 2500 using version 4 chemistry to achieve a minimum of 40 million 125 base read pairs. The data was processed using RTA version 1.18.61 and CASAVA 1.8.2.

### RNA-Sequencing data processing

General quality control checks were performed with FastQC v0.8.0 (31). Each dataset was filtered for average base quality score (>20). Multiple runs of the same sample library were merged prior to analysis. Filtered datasets were mapped against the mm10 reference genome using TopHat v2.0.12 (parameters–b2-sensitive–library-typefr-unstranded-g10–mate-innerdist-60–mate-std-dev 50) (32). Differential analysis at the gene level was performed with Cuffdiff v2.2.1 (33) at default parameters, using RefSeq gene model annotations downloaded from the UCSC Genome Browser as of March 9, 2015. Differentially expressed genes were identified using default Cuffdiff criteria (FDR < 0.05). Heatmaps were constructed with heatmap.2 (gplots R package) based on FPKM scores calculated by the Cuffquant/Cuffnorm v2.2.1 workflow. Assessment of repeat elements are based on RepeatMasker annotations downloaded from the UCSC Genome Browser (timestamp March 7, 2012). For identification of individual differentially expressed retrotransposons, only uniquely-mapped hits were retained for analysis. Mapped read counts for each annotated RepeatMasker element were collected with HTSeq-count^35^. The dataset was then reduced to only LINE, SINE, and LTR annotations with a minimum average depth of 1 read per replicate; the resulting data matrix included approximately 460k individual RepeatMasker annotations. DESeq2 DESeq2^36^ was then used to identify differentially expressed elements at an adjusted p-value threshold of 0.1.

### Statistical analysis

Data were analyzed using Prism (Version 6, GraphPad Software) by unpaired Student’s *t*-test with Welch’s correction and one-tailed p-value. Values are presented as mean ± s.e.m. A minimum of three biological replicates was used for each experiment.

### Data availability statement

The datasets generated during and/or analyzed during the current study are available in the GEO under accession GSE72808.

## Electronic supplementary material

Supplemental info
