# Supplementary material for: Loss of Glis3 causes dysregulation of retrotransposon silencing and germ cell demise in fetal mouse testis
Source: Sci Rep. 2018 Jun 25;8:9662. doi: 10.1038/s41598-018-27843-x (PMC6018429; doi:10.1038/s41598-018-27843-x)
Supplement: Supplementary file 1 — Supplemental info [file 41598_2018_27843_MOESM1_ESM.pdf]

Supplemental Figures & Tables

Loss of *Glis3* causes dysregulation of retrotransposon silencing and germ cell demise in fetal mouse testis

Erica K. Ungewitter[1], Emmi Rotgers[1], Hong Soon Kang[2], Kristin Lichti-Kaiser[2], Leping Li[3], Sara A. Grimm [4], Anton M. Jetten[2], and Humphrey H.-C. Yao[1*]

[1] Reproductive Developmental Biology Group, [2] Immunity, Inflammation & Disease Laboratory, and Biostatistics & Computational Biology Branch[3], Integrative Bioinformatics Support Group[4] , National Institute of Environmental Health Sciences, Research Triangle Park, NC, USA

Supplemental Materials & Methods

Differential motif enrichment analysis

We extracted the 1kb promoter sequences of the 128 differentially expressed genes using the mouse reference gene annotation (mm10). Since some of the genes contain multiple transcription start sites, the total number of unique promoter sequences identified was 144. We referred to those sequences as foreground sequences. For each promoter sequence of the differentially expressed genes, we randomly selected 20 promoter sequences of the same length (1kb) with similar C/G-content from the entire mouse genome as the matched background sequences. We then scanned the foreground promoter sequences and the corresponding background sequences for putative transcription factor binding sites using GADEM software (23). The motif models were obtained from TRANSFAC (24), JASPAR (25), and UniPROBE (26), The Glis3 motif model was derived from mouse ChIP-seq data generated in αTC-1 cells (see below). For the motif model, we counted the number of foreground sequences and the background sequences containing at least one predicted site for the motif. Motif enrichment analysis was carried out on the resultant 2×2 contingency table using Fisher exact test.

Supplemental Figure Legends

Supplemental Figure 1. Pure populations of germ and somatic cells isolated by fluorescence activated cell sorting of Oct4-eGFP testes. (A-B) Histograms of GFP fluorescence intensity (x axis) versus cell count (y axis) in WT (A) and E13.5 Oct4-eGFP mutant testes (B) at E13.5. (C) Scatter plot of cellular forward scatter (FSC-A, x axis) versus GFP fluorescence intensity (y axis) in E13.5 Oct4-eGFP testes cells. The gated cell

populations shown in aqua were reserved as our GFP Negative cells, while cells shown in green constitute the GFP positive cell fraction. (D-E) qPCR of somatic (*Sox9*, D) and germ (*Ddx4*, E) cell-specific genes in whole E13.5 testis (white bars) or the cellular fractions illustrated in panel C (aqua bars for GFP negative cells and green bars for GFP positive cells). Note that the GFP negative cell fraction is highly pure, with undetectable levels of the germ cell marker *Ddx4* (E). Expression levels of whole testis samples are set as 1 in (D-E).

Supplemental Figure 2. Global mRNA changes and pathway analysis. (A) A volcano plot of genes differentially expressed at E14.5 by RNA-Seq reveals a slight trend for genes to be downregulated (blue) over upregulated (red). (B-C) GO analysis was used to reveal the top 10 most affected biological processes in Glis3 testes at E14.5 and the differentially expressed genes involved in each process.

Supplemental Figure 3. Mutant germ cells do not precociously enter meiosis. Immunostaining of the germ cell marker TRA98 (red, A-C) or meiotic chromosome marker SYCP3 (D-F, green) in WT ovaries (A,D), WT testes (B,E), or Glis3 mutant testes (C,F) at E15.5. As expected for normal E15.5 gonads, SYCP3 positive meiotic germ cells are detected in the ovary (D) but not in the testis (E). Glis3 mutant testes do not contain SCYP3 positive germ cells (F). Scale bars: 200 μm in A & D, 100 μm for B, C, E, F.

Supplemental Figure 4. Somatic cell and pluripotency germ cell makers are not altered following ectopic expression of Glis3. (A) The targeting construct used to generate the Glis3+ mouse line. (B) Relative expression of somatic and germ cell genes in Glis3+ mutants (hot pink) or control testes (Glis3+, Cre -; pale pink) at E14.5. Expression of genes in WT testis are set as 1.

Supplemental Figure 5. Putative GLIS3 binding sites are enriched in the promoters of genes differentially expressed in Glis3 mutants. (A) A consensus GLIS3 motif logo was derived from αTC1 cell ChIP-seq data using GADEM software. (B) The promoter sequences of genes differentially expressed in Glis3 mutant testes were scanned for the GLIS3 motif. Putative GLIS3 binding sites (red text) were detected within 1 kb of the transcription start site of 24 unique germ cell related gene promoters. Several promoters (including Ddx4, Dnmt3l, Tdrd5, and others) contain multiple putative binding sites for GLIS3.

Supplemental Table 1. The top 10 enriched motifs in the promoters of differentially expressed genes belong to bHLH, Zinc finger and E2F families of transcription factor binding sites.

Supplemental Table 2. Probes and primer sets used for qPCR gene expression analysis.

Figure S1

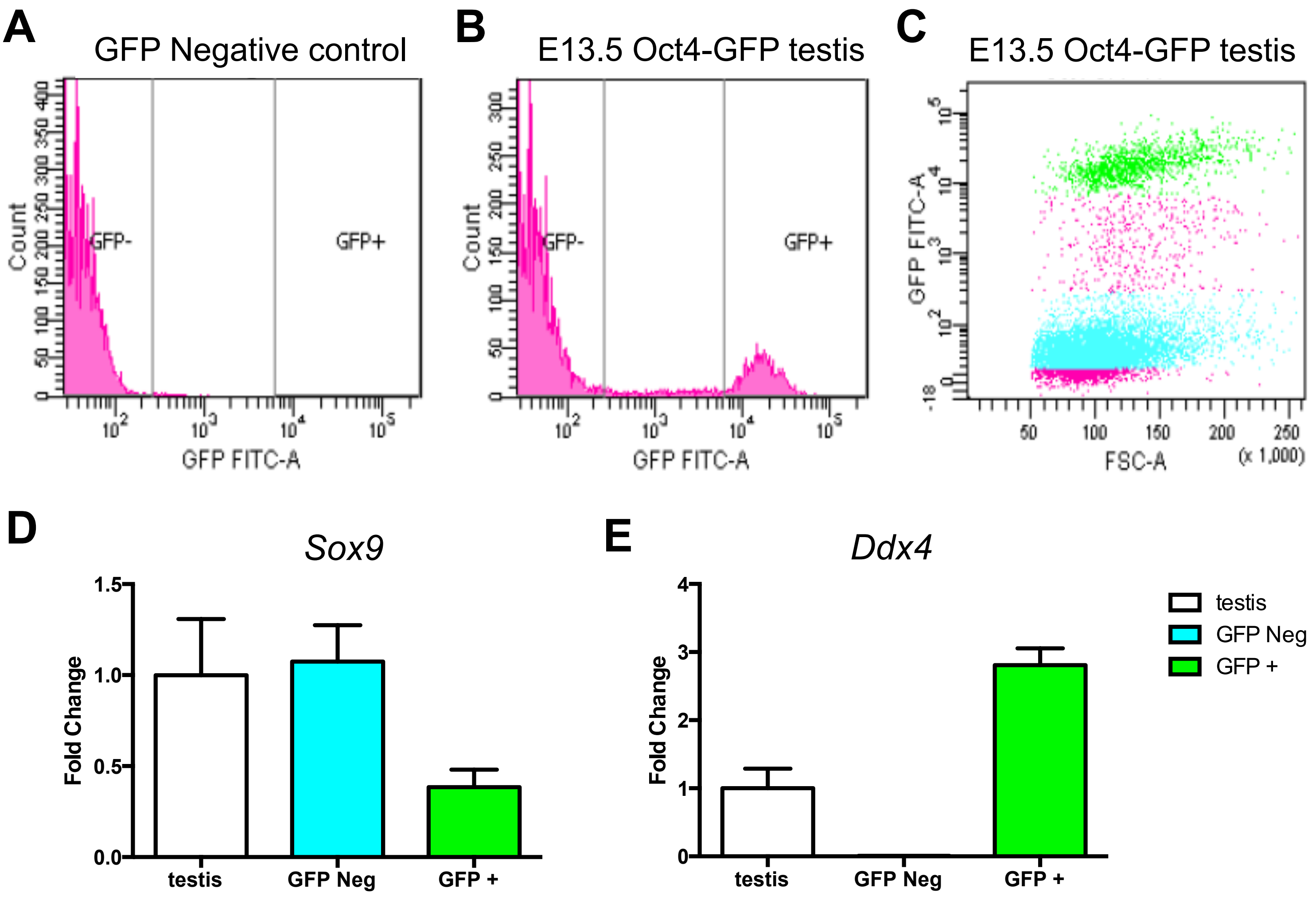

A
GFP Negative control
Count
GFP-
GFP+
GFP FITC-A
B
E13.5 Oct4-GFP testis
Count
GFP-
GFP+
GFP FITC-A
C
E13.5 Oct4-GFP testis
GFP FITC-A
FSC-A
(x 1,000)
D
Sox9
Fold Change
testis
GFP Neg
GFP +
E
Ddx4
Fold Change
testis
GFP Neg
GFP +
testis
GFP Neg
GFP +

Figure S2

A

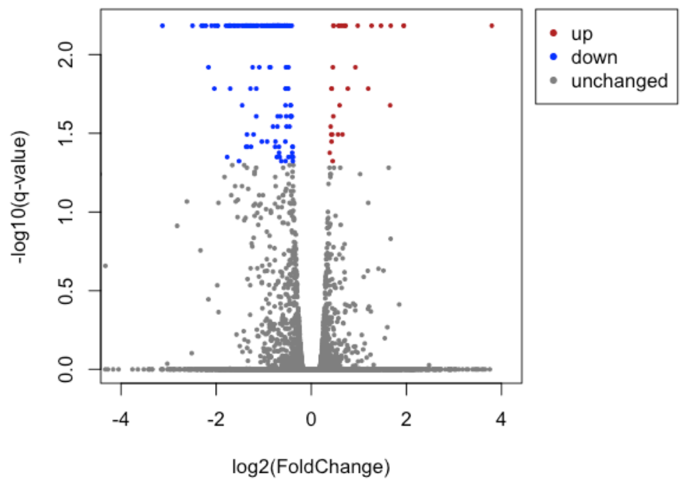

B

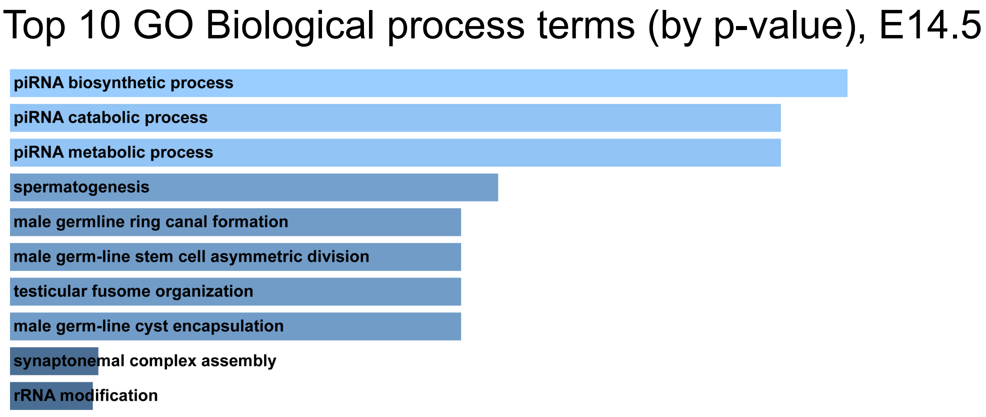

C

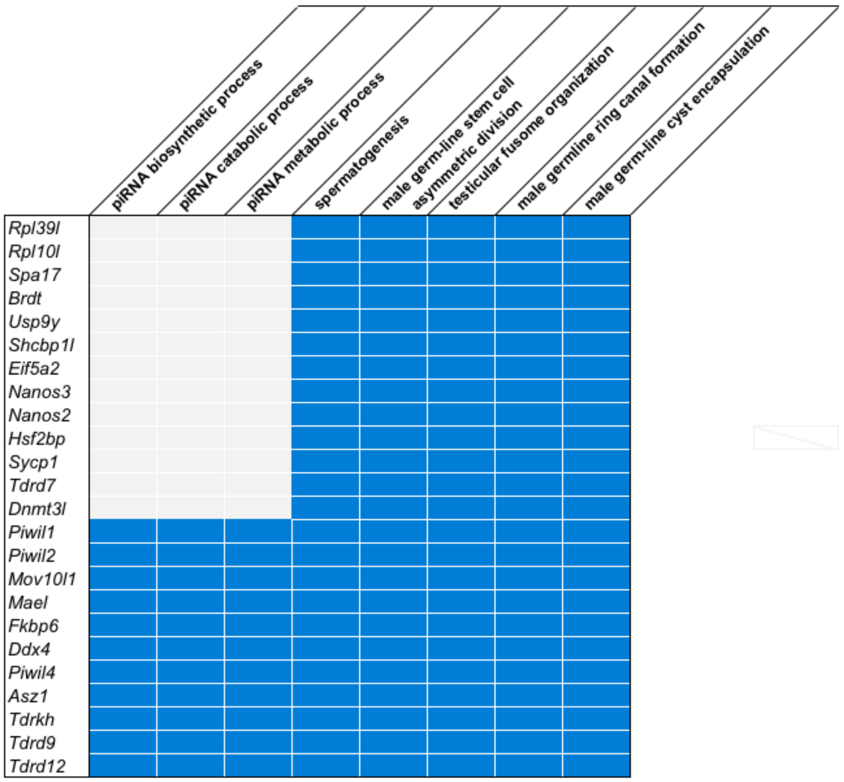

Figure S3

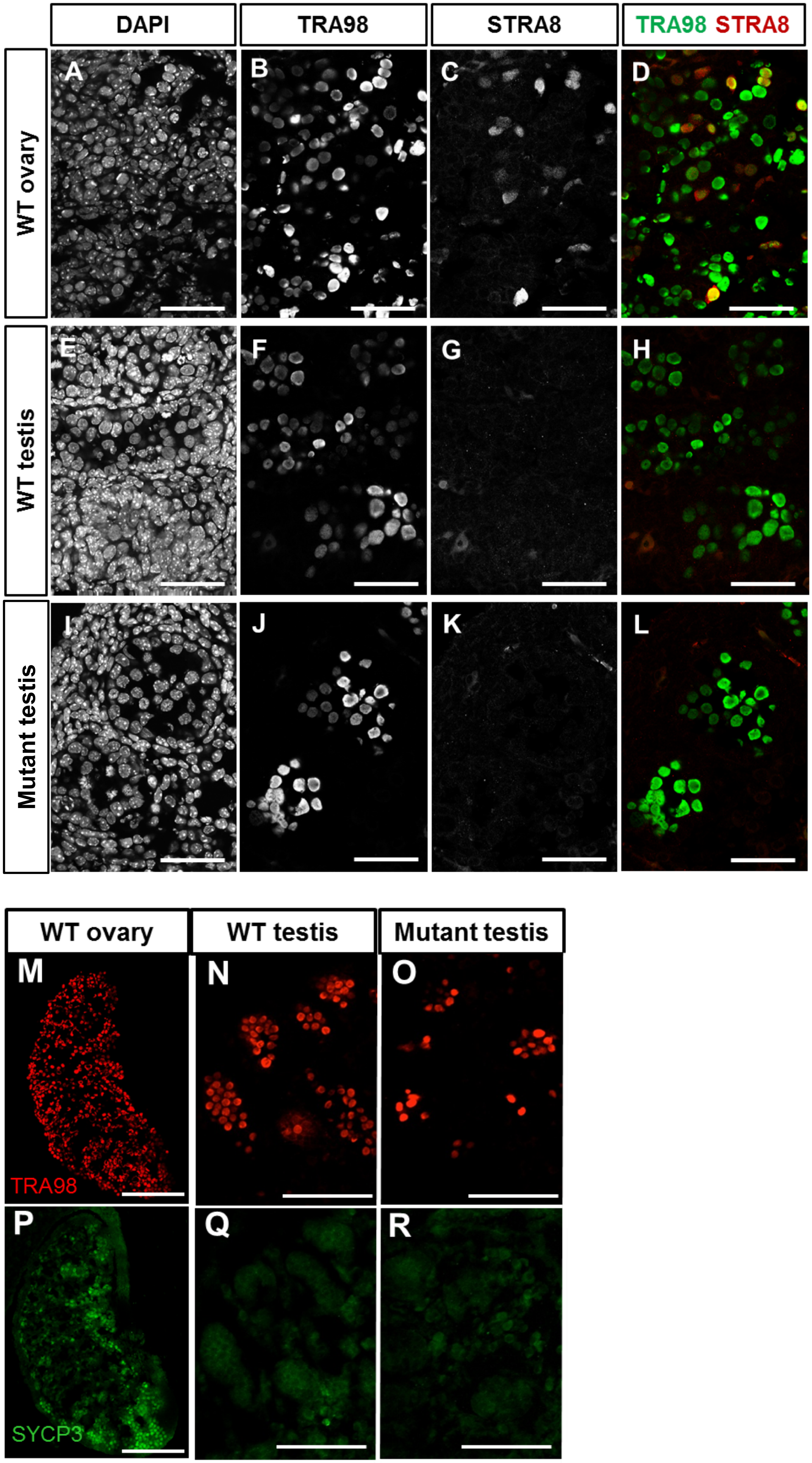

DAPI
TRA98
STRA8
TRA98 STRA8
WT ovary
WT testis
Mutant testis
A
B
C
D
E
F
G
H
I
J
K
L
WT ovary
WT testis
Mutant testis
M
N
O
TRA98
P
Q
R
SYCP3

Figure S4

A

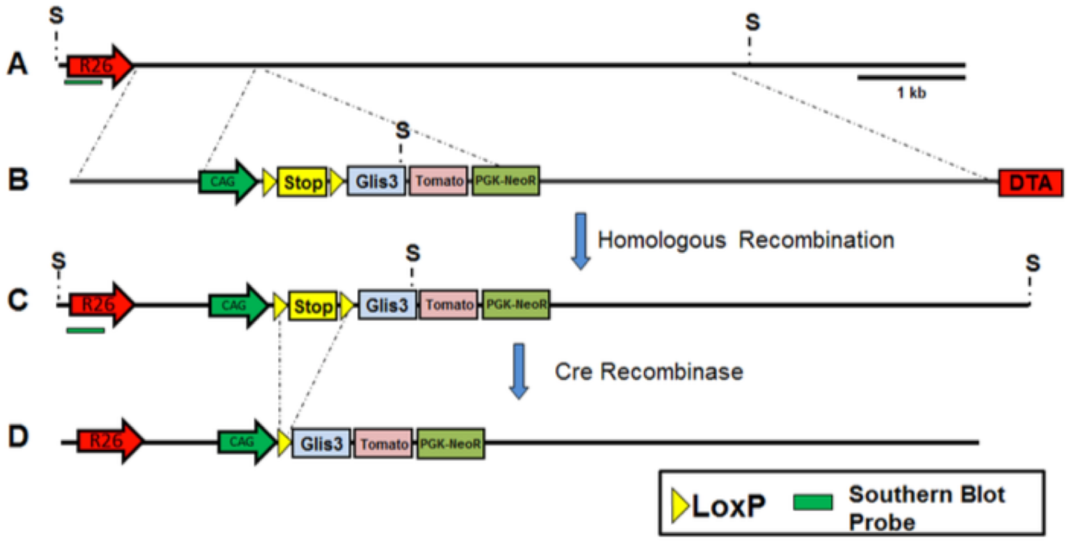

S
A
R26
S
1 kb
S
B
CAG
Stop
Glis3
Tomato
PGK-NeoR
DTA
Homologous Recombination
S
C
R26
CAG
Stop
Glis3
Tomato
PGK-NeoR
S
S
Cre Recombinase
D
R26
CAG
Glis3
Tomato
PGK-NeoR
LoxP
Southern Blot Probe

B

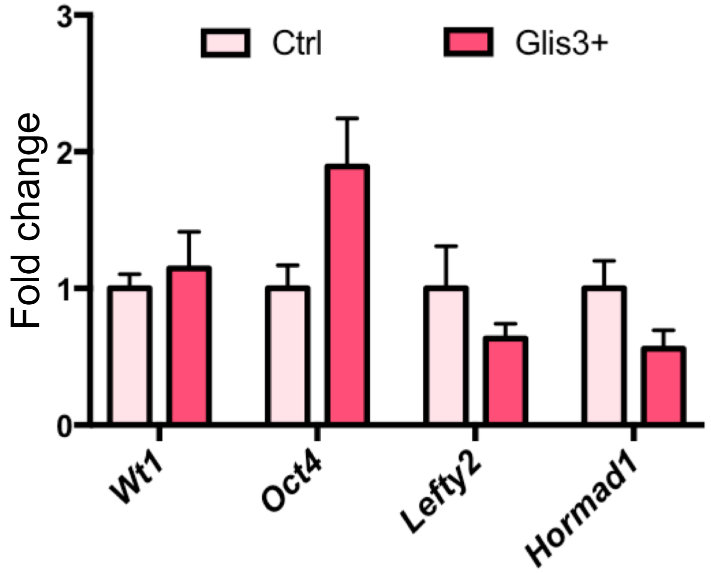

Fold change
0
1
2
3
Ctrl
Glis3+
Wt1
Oct4
Lefty2
Hormad1

Figure S5

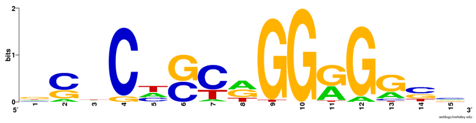


```
Ddx4     ---tgaGGCCTGTGGGAGTGAgct---
         ---ctgGGGCTCGGGGAGGCAgag---
Dnmt3l   ---ctgGGTCTCCCTGGCAGCctg---
         ---gtgGGGGCCCTGGAGGGGcag---
         ---tggACTGCCTAGGAGGGGggt---
         ---gccTCACAGTAGGAGGTAggg---
Tdrd5    ---gggACGCTCCGGGAGGGCcgc---
         ---aggCACCTGCGGGGGGCGggg---
         ---tagTGCCCCCCACCCCGCccc---
Mael     ---ttgGGCCCCCTGCTGTGGggg---
Morc1    ---tcgTGACCCCTGCAGATGgcg---
         ---tgaGAACACCAGGGGGCGaag---
Tdrkh    ---aaaCATCTCCTGGAGACTcgg---
         ---aggGGGCCCCTGCTGGCGcgc---
         ---aatTCTCAGCAGGTGCGAgct---
         ---gctGCGCTGAGGGAGCGAcag---
Piwil4   ---cccTGCCCCCCTCTCTGTcta---
Topaz1   ---gcaGGAGTGTTGGAGGCAgtt---
         ---gagGAACCCAAGGAGGCAcca---
         ---ctaGGCTTCCCAGAGAGGctg---
```

Supplemental Table 1. Top 10 enriched motifs in the promoters of genes differentially expressed in *Glis3* mutant testes at E14.5

TF family	Motif model	Motif logo	Model source	Enrichment *p*-value
bHLH	MYC_Q2	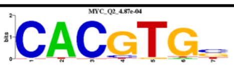	TRANSFAC	4.87e-04
Zinc finger	Sp4	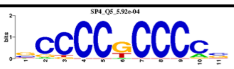	UniPROBE	5.92e-04
Zinc finger	WT1	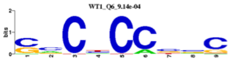	TRANFAC	9.14e-04
bHLH	ARH/ARNT	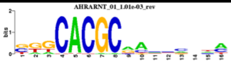	TRANFAC	1.01e-03
CCAT binding factor	NFYA	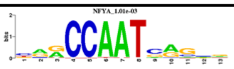	TRANFAC	1.01e-03
Zinc finger	**Glis3**	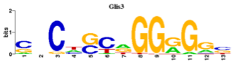	ChIP-seq derived	1.65e-03
Zinc finger	Zbtb7a	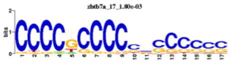	ChIP-seq derived	1.80e-03
E2F	E2F_02	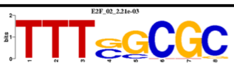	TRANSFAC	2.22e-03
E2F	E2F1DP1RB	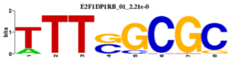	TRANSFAC	2.22e-03
bHLH	Mycn	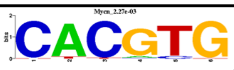	JASPAR	2.27e-03

Supplemental Table 2. qPCR primer sequences and probe information

SYBR based gene expression assays			
	(5' to 3', sequence)		
**Gene**	**Forward primer**	**Reverse primer**	**reference**
Ddx4	GAGAACACATCTACAACTGGTGG	AAACCTCTGTTTCCAAAGCCC	Harvard PrimerBank
Gapdh	TTCACCACCATGGAGAAGGC	GGCATGGACTGTGGTCATGA	Harvard PrimerBank
Glis3	CCATGTCCACGGATTTTATGG	CACAGTTCTTTGGGAATCAGGAT	(14)
IAPGag	AACCAATGCTAATTTCACCTTGGT	GCCAATCAGCAGGCGTTAGT	(19)
L1ORF2	GGAGGGACATTTCATTCTCATCA	GCTGCTCTTGTATTTGGAGCATAGA	(19)
Mapk1	CCTTCAGAGCACTCCAGAAAGT	ACAACACCAAAAAGGCATCC	(36)
Neurog3	TTCTTTTGAGTCGGGAGAACTAGG	GGGACACTTGGATGGTGAGC	(14)
Pou5f1	ACCTGGCTTCAGACTTCGC	TGAGCCTGGTCCGATTCCA	Harvard PrimerBank
Rec8	CTACCTAGCTTGCTTCTTCCCA	GCCTCTAAAAGGTGTCGAATCTG	(37)
Sox9	AAGAAAGACCACCCCGATTACA	CAGCGCCTTGAAGATAGCATT	Harvard PrimerBank
Stra8	GCCGGACCTCATGGAATTTGA	TCACTTCATGTGCAGAGATGATG	Harvard PrimerBank

Taqman gene expression assays	
**Gene**	**Probe reference #**
Boll	Mm01255492_m1
Cyp11a1	Mm00490735_m1
Dnmt3l	Mm00457635_m1
Gapdh	mm99999915_g1
Hormad1	Mm00471448_m1
Lefty2	Mm0077457_m1
Mael	Mm01293195_m1
Oct4	Mm00658129_gH
Piwil1	Mm01204593_m1
Piwil2	Mm00502383_m1
Piwil4	Mm01144775_m1
Tdrd1	Mm00459548_m1
